# Testing a pain self-management intervention by exploring reduction of analgesics’ side effects in cancer outpatients and the involvement of family caregivers: a study protocol (PEINCA-FAM)

**DOI:** 10.1186/s12912-018-0323-x

**Published:** 2018-12-12

**Authors:** Sabine Valenta, Rebecca Spirig, Christine Miaskowski, Kathrin Zaugg, Elisabeth Spichiger

**Affiliations:** 1 Nursing Science, Department Public Health, University of Basel, Bernoullistrasse 28, CH-4056 Basel, Switzerland; 2 Department of Hematology, University Hospital Basel, Basel, Switzerland; 3 Department of Nursing Science, University of Witten/ Herdecke, Witten, Germany; 4 School of Nursing, University of California San Francisco (UCSF), San Francisco, USA; 5 Department of Radiation Oncology, Stadtspital Triemli, Zurich, Switzerland; 6 Department of Radiation Oncology, Inselspital, University Hospital Bern, University of Bern, Bern, Switzerland; 7 Head Office of Nursing and Allied Health Professionals, Inselspital, University Hospital Bern, Bern, Switzerland

**Keywords:** Neoplasms, Pain management, Adverse effects, Caregivers, Health behaviour, Self-care, Patient education, Randomized controlled trial, Health knowledge, attitudes, practice

## Abstract

**Background:**

Pain is one of cancer patients’ most frequent and distressing symptoms; however, analgesics’ side effects often increase symptom burden. Further, with the home rapidly becoming the primary cancer care setting, family caregivers (FCs) commonly play central roles in patients’ pain self-management, but with little or no preparation. One US-tested intervention, the PRO-SELF© Plus Pain Control Program (PCP), designed to support cancer outpatients and their FCs in pain self-management, is currently being tested in the Swiss multi-centre PEINCA study. The current PEINCA-FAM study is a sub-study of PEINCA. The aims of PEINCA-FAM are: a) to test the efficacy of the adapted German PRO-SELF © Plus PCP to reduce side effects of analgesics; b) to enhance patients’/FCs’ knowledge regarding cancer pain; and c) to explore FCs’ involvement in patients’ pain self-management.

**Methods:**

This mixed methods project combines a multi-centre randomized controlled clinical trial with qualitative data collection techniques and includes 210 patients recruited from three oncology outpatient clinics. FCs involved in patients’ pain self-management are also invited to participate. After baseline evaluation, eligible participants are randomized to a 6-week intervention group and a control group. Both groups complete a daily pain and symptom diary. Intervention group patients/FCs receive the weekly psychoeducational PRO-SELF© Plus PCP interventions; control group patients receive usual care. After completing the six-week study procedures, a subsample of 7–10 patients/FCs per group and hospital (*N* = 42–60) will be interviewed regarding their pain management experiences. Data collection will take place from April 2016 until December 2018. An intent-to-treat analysis and generalized linear mixed models will be applied. Qualitative data will be analysed by using interpretive description. Quantitative and qualitative results will be combined within a mixed method matrix.

**Discussion:**

In clinical practice, specially trained oncology nurses in outpatient clinics could apply the intervention to reduce side effects and to enhance patients’/FCs’ self-efficacy and pain management knowledge.

**Trial registration:**

The PEINCA study is registered in the Clinical Trials.gov site (code: NCT02713919, 08 March 2016).

## Background

### Cancer pain and analgesics’ side effects management

Pain is one of cancer patients’ most frequent and distressing symptoms. However, analgesics’ side effects often increase symptom burden [1, 2]. The International Association for the Study of Pain defines pain as “an unpleasant sensory and emotional experience associated with actual or potential tissue damage, or described in terms of such damage” [3]. World Health Organization (WHO) guidelines note that the main focus of pain management is analgesics. As depicted in Table 1, these guidelines focus on a 3-step progression of analgesics from non-opioids to strong opioids, which are associated with burdensome side effects [4, 5]. For opioids, the most common of these side effects are constipation, nausea, emesis, and concentration difficulties; the most feared, though very rare, are respiratory depression and death. However slight the risk, fear may still lead to early discontinuation, underdosing, or otherwise inadequate analgesia [6, 7].

**Table 1:** Categorization of pain, appropriate analgesics, side effects, and co-medication

WHO analgesic ladder steps	Score 0/10 on NRS	Category of analgesic	Most important analgesic	Most important side effects	Co-medication
1 (mild pain)	< 3	Non-opioids	- Nonsteroidal Anti-Inflammatory Drugs (NSAIDs)	- gastrointestinal complications - heart attack - stroke - skin or allergic reactions	- Proton pump inhibitor - Antihistamines - Corticosteroids
			- Acetaminophen - Metamizole	- renal and liver toxicities - Hepatotoxicity - skin or allergic reactions	- Antihistamines - Corticosteroids
2 (mild to moderate pain)	3–6	Weak opioids ± Non-opioids	- Tramadol - Tilidine + Naloxone	- Constipation - Nausea - Pruritus - motor and cognitive impairment - respiratory depression - sedation	- Laxatives - Anti-emetics - Corticosteroids - Antihistamines - Major tranquillizers - Psychostimulants
3 (moderate to severe pain)	> 6	Strong opioids ± Non-opioids	- Morphine - Hydromorphone - Oxycodone - Methadone - Levorphanol - Pethidine		

Note. Categorization of pain, appropriate analgesia, side effects and co-medication according to the WHO-sequential three-step analgesic ladder [5–7, 63, 70, 71]

Abbreviations: *WHO* World health organization, *NRS* Numeric rating scale, *NSAIDs* Nonsteroidal anti-inflammatory drugs

Although pain is a treatable symptom, up to 40% of cancer patients experience inadequate pain management. System-level barriers include limited access to pain specialists or opioids [8] and misconceptions of healthcare professionals (e.g., misconceptions and knowledge gaps concerning pain management [9]). Patient-level barriers can be divided into four groups: cognitive (e.g., lack of information), affective (e.g., stress, anxiety, depression), sensory (e.g., analgesics’ side effects) and practical (e.g., implementing pain management in everyday life) [10]. Family caregivers (FCs) directly involved in patients’ pain management commonly experience the same barriers as the patient. FCs are defined as relatives, partners, friends, or neighbours who provide care to patients [11].

With the home becoming the primary setting for many aspects of cancer care, a key goal is to improve patients’ pain and side effect self-management [12]. Self-management consists largely of strategies for solving problems, making decisions, taking action, utilizing resources, forming patient/healthcare provider partnerships, and dealing with physical and psychological issues to avoid or delay deterioration [13, 14]. FCs commonly play central roles in patients’ pain self-management. However, while FCs may administer medications, implement pain relief strategies, and provide emotional support [10, 15], they often assume their tasks with little or no preparation, knowledge, resources, or skills [16]. The psychological and physical costs include a high prevalence of anxiety and depression, social isolation, sleep disturbances, fatigue, and even an elevated risk of coronary heart disease [17–19]. The responsibility for pain management without corresponding skills often results in FCs’ loss of self-efficacy [20]. Self-efficacy is defined as the confidence in one’s ability to perform the behaviours necessary to achieve a target outcome [21]. Recent studies on cancer pain management reveal that high self-efficacy improves both patients’ well-being and FCs’ mood [22, 23]. However, little research has focused on FCs’ influence and self-efficacy vis à vis supporting self-management of pain and analgesics’ side effects in cancer outpatients.

### Supporting self-management of pain and analgesics’ side effects

Three recent meta-analyses focusing on the effects of psychosocial interventions to support cancer pain management from 1983 to 2012 found that, while gaining attention, the psychosocial approach to pain management remains understudied [24–26]. The two most common intervention types were skill training and education [25]. Regarding the main outcome measures, i.e., effects on pain intensity, pain interference in daily activities, knowledge, and attitudes towards cancer pain and analgesics, these meta-analyses revealed various significant, though moderate overall improvements. Further, a systematic evaluation of interventions’ content, structure, and efficacy to improve patients’ self-management of cancer pain revealed no discernible patterns with respect to components, duration, or delivery type or mode [27]. We found no studies that focused on the side effects of analgesics.

To date, little research has focused on interventions supporting FCs in patients’ cancer pain self-management. First, Keefe et al. [28] tested the feasibility of a partner-guided cancer pain management training program with 78 end-of-life patients and their FCs. Intervention group FCs showed significantly higher levels of self-efficacy regarding support for the control of pain and other symptoms. Second, in a randomized controlled trial (RCT) of caregiver assisted coping skill training for cancer patients and their FCs, Porter et al. [29] reported significant results compared with an education program. Additionally, enhanced knowledge of cancer pain management led to both decreased anxiety symptoms in both groups and increased symptom management self-efficacy in FCs. However, as this study lacked a standard care control condition, these results should be interpreted with caution. Third, Hendrix et al. [23] tested an individualized training intervention on self-efficacy in 120 patient-caregiver dyads. FCs in the intervention group demonstrated significant increases in self-efficacy regarding pain control, prevention of infections, maintenance of nutrition, and practical home care issues.

However, no studies have evaluated the effectiveness of interventions focusing on analgesic side effect management to improve self-management in cancer patients and their FCs. Similarly, no study has yet investigated correlations between FCs’ cancer pain management knowledge and self-efficacy related to pain management and patients’ pain intensity.

### The PRO-SELF© plus pain control program, PEINCA and PEINCA-FAM

One psychoeducational intervention included in the above-noted meta-analyses, the PRO-SELF © Pain Control Program (PCP), an intervention to support self-management of pain in adult oncology outpatients and their FCs, showed statistically significant and clinically meaningful reductions compared to standard care in both average and worst-pain intensity in a large RCT (*N* = 174) [30]. In further research, the intervention was expanded regarding its duration (from 6 to 10 weeks) resulting in the PRO–SELF © Plus PCP [31, 32]. Koller, Miaskowski, De Geest, Opitz, and Spichiger [33] translated and adapted this version for use in German-speaking populations. A pilot RCT using 39 oncology outpatients yielded a statistically significant increase in test subjects’ knowledge and demonstrated this version’s feasibility. Based on the pilot RCT’s findings, the intervention was adapted and is currently being tested within the multi-centre “Mixed methods study to test the efficacy of the adapted German PRO–SELF © Plus PCP, an intervention directed at outpatients with cancer and their family caregivers to reduce pain and related symptoms” (PEINCA) in Switzerland. The overall aim of the multi-centre mixed methods PEINCA study is to evaluate the efficacy of the adapted German PRO-SELF© Plus PCP, which was designed to improve outpatients’ and their FCs’ management of pain and pain intensity. The purpose of this sub-study of PEINCA is to test the efficacy of the adapted German PRO-SELF © Plus PCP at both reducing analgesic side effects and enhancing cancer patients’ and FCs’ knowledge of cancer pain as well as to explore their learning processes. In addition, FCs’ involvement in patients’ pain self-management and association between their self-efficacy and knowledge of cancer pain with the cancer patients’ pain intensity will be investigated (PEINCA-FAM).

### Theoretical framework

**Fig. 1:**
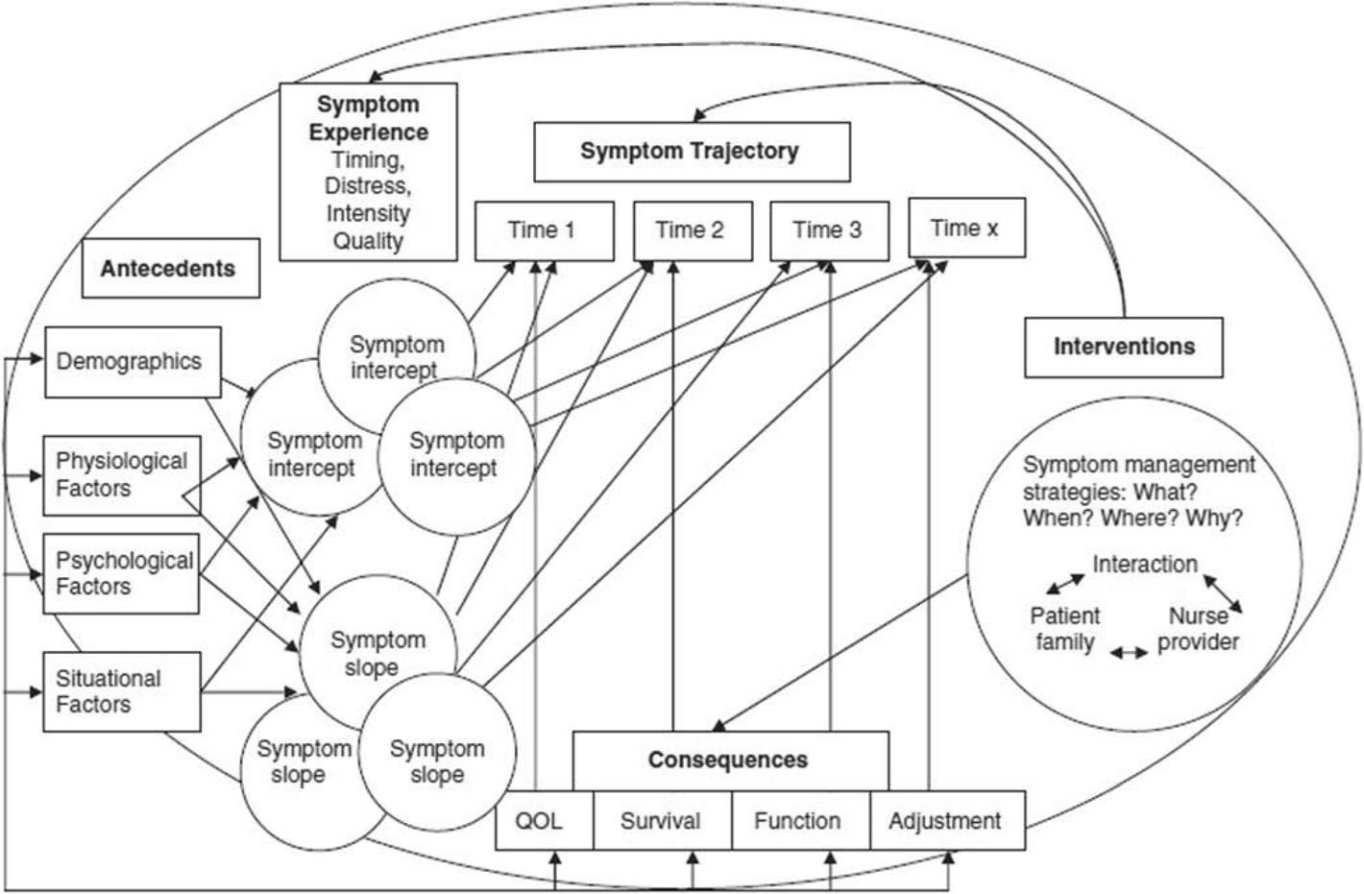
Diagram of the Symptom Management Model. *Note.* The Symptom Management Model (SMM) [35]. Abbreviation: QoL = quality of life. Copyright © gratefully received by Copyright Clearance Center’s RightsLink® service/ John Wiley and Sons

The overall framework of the PRO-SELF © Plus PCP was the Theory of Symptom Management (TSM) [34]. A further development of the TSM, the Symptom Management Model (SMM, see Fig. 1), which depicts interacting concepts, serves as the theoretical framework for this study [35]. Precipitating factors (antecedents) such as demographic, sociocultural and psychological characteristics, can influence perceived symptoms at baseline (intercepts) and over a given trajectory (slope). By charting the trajectories of symptoms in relation to interventions (symptom management strategies) and patient/FC/nurse interactions, we can illustrate these interactions’ continued influence over patients’ and their FCs’ outcomes (consequences). In this study, consistent with the SMM, cancer pain is viewed as a complex, multidimensional experience frequently accompanied by analgesics’ side effects. Precipitating factors, e.g., social environments and lack of knowledge, influence symptom trajectories. Within the framework’s symptom management strategy, our intervention focuses on supporting cancer patients’ pain and analgesic side effect self-management and the involvement of FCs. Patients as well as FCs are seen as key players who interact with intervention nurses. Alongside improved knowledge of cancer pain and self-efficacy in FCs, reduction of analgesics’ side effects is a key intervention outcome.

In addition, Bandura’s Social Cognitive Theory (SCT), an adult learning theory, provided the supplementary conceptual framework for the intervention [36]. SCT acknowledges that learning is a cognitive process that takes place in a social context and can occur through processes of observation, with each individual possessing a self-regulating system that affects motivation and learner differentiation. As part of this system, Bandura introduced the concept of self-efficacy [37], along with major influencing factors: mastery experiences, live modelling, performance exposure and positive appraisal [21]. Through our intervention to educate cancer patients and their FCs, we aim to develop their knowledge regarding pain management, their self-efficacy regarding pain management, and their symptom self-management skills. This entails keeping a diary, setting goals and establishing a management plan to reach those goals.

### Aims

PEINCA-FAM aims to test the adapted German PRO-SELF © Plus PCP pain self-management intervention to reduce cancer outpatients’ analgesics’ side effects, to explore patients’ and FCs’ knowledge of cancer pain and their learning processes, and to investigate FCs involvement in patients’ pain self-management and associations between their self-efficacy, knowledge of cancer pain and patients’ pain intensity. Specific aims of this study are: (1) to test the efficacy of the adapted German PRO-SELF © Plus PCP to reduce analgesics’ side effects by focusing on constipation, nausea, emesis and concentration difficulties; (2) to test the efficacy of the adapted German PRO-SELF © Plus PCP to improve knowledge of cancer pain management in patients and FCs and to qualitatively explore patients’ and FCs’ learning process during the intervention; and (3) to explore the association between FCs’ self-efficacy and knowledge of cancer pain with patients’ pain intensity and to qualitatively explore FCs’ influence on patients’ pain self-management.

## Methods

### Study design

Embedded in the ongoing PEINCA study, the PEINCA-FAM sub-study applies a mixed method approach. In a concurrent embedded strategy, an RCT is combined with qualitative parts [38, 39]. All data are generated within PEINCA. The following describes the PEINCA study’s essential methods as they apply to PEINCA-FAM.

### Sample and setting

Patients with cancer pain are recruited from oncology outpatient clinics at three university hospitals in the German speaking part of Switzerland. These individuals are included if they (1) are aged ≥18 years; (2) have experienced any type of recurrent cancer pain, i.e., rated ≥3 on a 0–10 Numeric Rating Scale (NRS, 0 = no pain, 10 = worst imaginable) over the past week; (3) have an estimated life expectancy of > 6 months; (4) are able to understand, read and write German; and (5) have access to a telephone. Participants are excluded if they (1) have cognitive dysfunction or hearing impairment; (2) are hospitalized > 2 weeks during the study; and (3) are suffering solely from neuropathic pain. To be eligible, FCs must be (1) aged ≥18 years; (2) able to understand, read and write German; and (3) willing to participate in all intervention sessions.

### Sample size determination

The results of the above-mentioned pilot study (Koller et al. [33]) included a maximum mean alleviation of 0.99 NRS (0–10) for average pain and a mean alleviation of 1.27 NRS (0–10) for the worst pain compared to the control group in week nine. For these two endpoints, based on average pain improvement data, an alpha error of 0.05 (95%) and a beta error of 0.2 (80%) would require a 136-patient sample to achieve sufficient statistical power. Estimating an attrition rate of 35% over the 6-week study period, it will be necessary to recruit a total sample of 210 patients. We expect to recruit 50% of patients along with FCs. This number will be sufficient. Based on Wells, Hepworth, Murphy, Wujcik, and Johnson [40], who assessed initial effects of an intervention to increase knowledge and positive beliefs about cancer pain management in patients using the Family Pain Questionnaire (FPQ), an improvement in knowledge and beliefs were found [F (1,62) = 18.2, *p* < 0.001]. At an alpha error level of 0.05, a sample size of 32 FCs should be sufficient to achieve a statistical power of 80%.

With respect to the sample size for the qualitative part, a sample of 42–60 patients and FCs should supply sufficiently redundant data to discover patterns, commonalities and differences among the groups.

### Recruitment

Specifically trained nurses from the outpatient clinics work as research assistants (RAs) for the study. All RAs are experienced registered nurses or graduate students in nursing who have received study-specific training by the study coordinator (HR). RAs screen potential participants and verify inclusion criteria via patient files and discussions (regarding life expectancy, etc.) with their treating physicians. If the initial inclusion criteria are met, the RAs contact the patients directly during their outpatient appointment to check whether they have experienced repeated pain that they would rate on an NRS as ≥3 over the last week, are able to understand, read and write German, and have access to a telephone. The RAs then inform patients about the study and invite them to participate. Each patient is also asked if an FC is involved in their daily pain self-management. If yes, the FC is also invited to participate in the study. Written information and consent forms (including those for FCs, if necessary) are then given to the patient. After at least 24 h, an RA contacts each patient (or patient/FC dyad) again to provide verbal information to the FC as needed, ask about their decision regarding participation, and collect the signed written consent form. In all settings, these written informed consent forms are stored safely in the Investigator Site File (ISF) by the RAs. Only afterwards are participants randomized to the IG or CG. Patients and FCs have the right to withdraw from the study at any time without consequences.

Concerning the recruitment of participants for qualitative data collection, all patients (and FCs) are asked at the beginning of the main study whether they are willing to participate in an interview following completion of the study’s 6 weeks of procedures. At the end of the final home visit, the intervention nurse (IN) or an RA asks selected patients (and FCs) whether they are still willing to participate in the interview. They then provide verbal and written information and obtain written consent from those who agree. Purposive sampling is applied to ensure approximately equal sample sizes per study site, as well as variation regarding pain intensity, intervention adherence, age, gender, education, living situation, and tumour entity.

### Random assignment

**Fig. 2:**
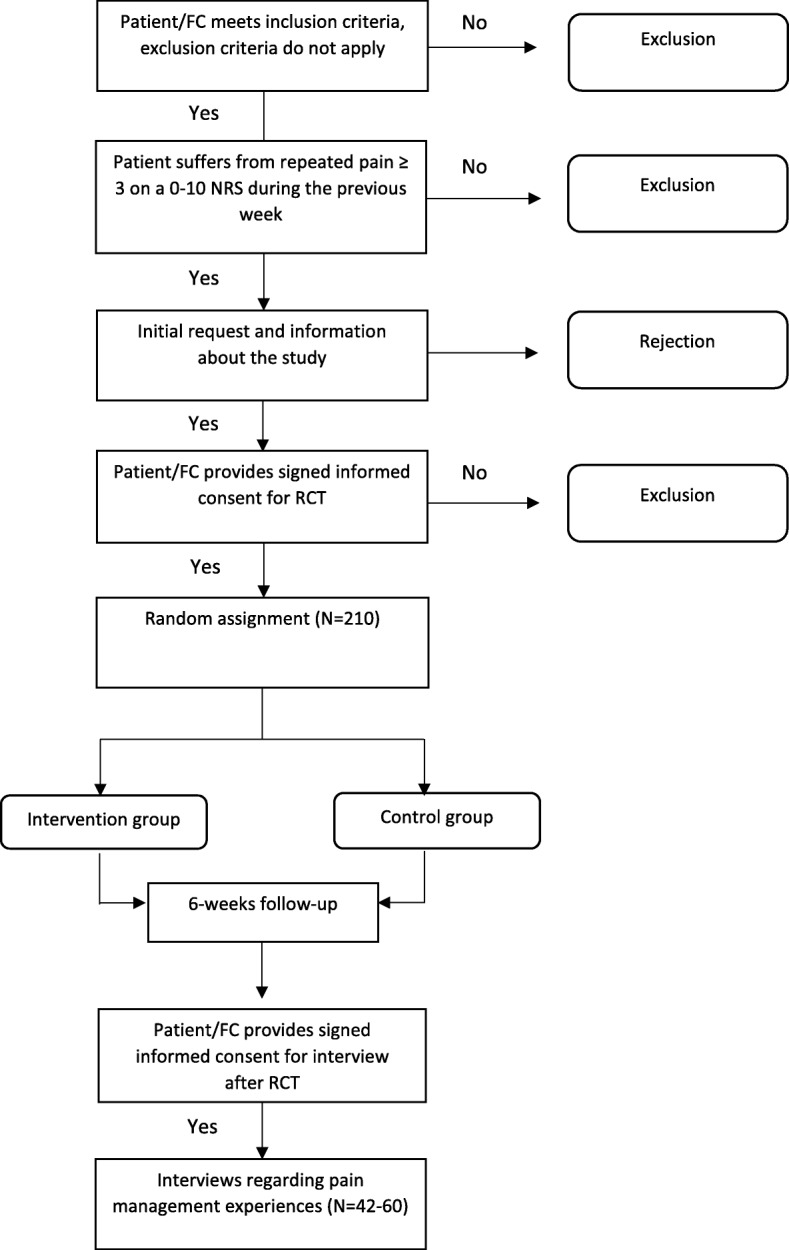
Flow chart of patient recruitment. Note. Flow chart of patient recruitment

As shown in Fig. 2, patients and FCs who have provided written consent are stratified by site and randomized 1:1 either to a six-week intervention group (IG) or to a usual care group (CG). A permuted block procedure, with blocks of 2, 4 and 6, is used to create a computer generated randomization list. This procedure should ensure approximately equal distribution of patients per group and per centre. The Clinical Trial Unit (CTU) of one of the included university hospitals provides the list, which is accessible from all settings.

### Blinding

Blinding is not possible because the INs use collected data directly for the intervention (pain diary, Patient Pain Questionnaire (PPQ)). And while treating physicians are not informed, they may still become aware of group allocation if participants ask questions or take their diary to an appointment.

### Intervention group and usual care group

#### Intervention group

The intervention is performed by four specially trained oncology nurses. These INs have completed a Master’s degree in nursing and are experienced clinicians in this field of study. A two-day training segment provided by the primary investigator (ES) included a review of current pain management guidelines and detailed teaching and training for each intervention component of the adapted German PRO-SELF © Plus PCP.

The intervention is intended to implement structured and tailored components of the German PRO-SELF© Plus PCP and is based on three key strategies: nurse coaching, skill building, and provision of information via academic detailing [30, 41]. Academic detailing focuses on enhancing baseline knowledge by providing key information and positive reinforcement, while stimulating the learner as an active partner [42, 43]. Structured intervention components focus on patients’/FCs’ education to monitor pain and analgesics’ side effects (e.g., nausea, emesis, constipation, concentration difficulties, fatigue), to document analgesics taken, and to use a one-week pillbox. They are also trained to apply a script to communicate with clinicians when pain control is inadequate.

To identify patients’ and FCs’ pain management knowledge deficits, the INs check their baseline knowledge of cancer pain and side effect management based on the results of the PPQ. To reinforce their education and enhance knowledge of cancer pain and side effects, patients and FCs receive the PRO-SELF © Pain Control Booklet, as well as individualized information.

Concerning the tailored intervention components, the IN reviews each pain diary with the associated patient/FC, assesses the appropriateness of the analgesic prescription and the side effect management, and teaches the patient/FC how to adjust the prescribed analgesics in response to changing pain conditions and side effects. Adherence to analgesic medication instructions and the IN’s recommendations is assessed and discussed with the patient/FC at each subsequent visit, after which modifications to the pain and side effect management plan are made as needed. The intervention is provided during in-home visits (baseline, weeks 1 and 6), followed by weekly in-home or telephone visits. The number, type (in-home visit or phone call) and frequency of visits are determined by defined criteria as follows: pain score > 3 on an NRS, patient is dissatisfied with pain management, patient adherence with pain medication or recommendations < 50%. If one or more of these criteria apply, the IN schedules an in-home visit; if none apply, she schedules a phone call. In addition, a reinforcement phone call is scheduled after major changes to the pain management plan.

For quality assurance and analysis, all intervention sessions and phone calls are audio recorded. The primary investigator and the study coordinator review audio recordings of visits and report their observations using an audit checklist based on the intervention protocol. In order to enhance the intervention’s implementation, they and the INs promptly discuss any deviations. They continue to monitor audiotapes until a 95% protocol adherence rate is achieved. Afterwards, audio-recordings are reviewed at random.

#### Usual care group

The CG participants receive usual care regarding pain management by participating centres. The treating physicians assess pain and prescribe analgesics. However, if participants raise concerns to RAs about pain or side effects, they are encouraged to contact their physicians. No specific counselling is provided.

### Data collection

Both quantitative and qualitative data will be collected from March 2016 until December 2018. Patients in both groups complete a daily pain and symptom diary; patients and FCs fill in questionnaires. The same 6-week data collection protocol is applied via in-home visits at baseline and during weeks 1 and 6. In the IG, data are collected by the IN. In order to minimize diffusion of the intervention to CG participants, an RA collects data in the control group. Between in-home visits at baseline and weeks 1 and 6, patients/FCs receive brief telephone calls every second week, during which the RA ensures that the pain diary is being completed on a daily basis.

### Quantitative data: variables and measurement

Table 2 provides an overview of the study variables and data collection points for patients and FCs.

**Table 2:** Study variables and measurement timetable

Study variable	Instrument	Assessed in patients	Assessed in FCs	Week 0	Week 1	Week 2–5	Week 6
Demographics, patient	Patient Information Questionnaire	X		X			
Demographics, FC	FC Information Questionnaire		X	X			
Clinical data	Medical Record Review Form	X		X			
Average pain and worst pain	Pain management diary^a:^ Brief Pain Inventory (BPI)	X		X	X	X	X
Pain alleviation through pain medication		X		X	X	X	X
Pain interference with function		X		X	X	X	X
Duration of pain Bowel movements and use of laxatives Side-effects of pain and cancer treatment	Pain management diary^a:^	X		X	X	X	X
Knowledge of cancer pain	Patient Pain Questionnaire	X		X			X
	Family Pain Questionnaire		X	X			X
Constipation	Constipation Assessment Scale	X		X	X	X	X
Self-efficacy	Self-Efficacy Questionnaire in patients with cancer	X		X			X
	Caregiver version of the Self-Efficacy Questionnaire		X	X			X
Anxiety and depression	Hospital Anxiety and Depression Scale	X		X			X
Functional status	Eastern Cooperative Oncology Group Performance Status	X		X			X

Note. ^a^Pain management diary: daily assessment of average and worst pain, pain alleviation, and pain interference with function via *BPI* Brief pain inventory as well as duration of pain, bowel movements/ use of laxatives, side effects of pain and of cancer treatment via pain management diary

Questionnaires employed in this study are applied in their German versions, for which validity and reliability have been established. The primary patient outcomes are selected analgesics’ side effects (constipation, nausea, emesis, and concentration difficulties), self-efficacy, and knowledge of cancer pain management. Primary FC outcomes are self-efficacy and knowledge of cancer pain management. The secondary outcomes are patients’ average and worst-pain intensity. Anxiety, depression, functional status, and cancer-related symptoms are measured as covariates. Our analytical models will also control for age, education, time since diagnosis and current disease status.

#### Medical record review form

Clinical data from patients’ medical records include the date of the initial diagnosis, type of tumour, existence of any metastases (no/yes regarding localization of metastases), comorbidities, and completed or current tumour therapy.

#### Patient/FC information questionnaires

These questionnaires assess socio-demographic data including age, sex, living alone (yes/no), employment status and educational level.

#### Pain management diary

A pain management diary is used to assess analgesics’ side effects for each 7-day period. Patients are asked to rate 12 side effect items on an 11-point NRS (0–10: 0 = no symptom experience; 10 = strongest imaginable symptom experience). Our analysis will focus on constipation, nausea, emesis, and concentration difficulties. On a second 11-point NRS (0 = no pain; 10 = strongest imaginable pain) patients also rate their daily average and worst pain intensity.

#### Brief pain inventory

Pain intensity, i.e., average and worst pain, is measured on 0–10-point NRSs, which are part of the German version of the BPI. Daily pain scores will be averaged for each week of the study. The BPI is a pain measurement tool with reliability and validity established as excellent [44, 45].

#### Constipation assessment scale (CAS)

The CAS, an 8-item self-report tool, is completed weekly to measure the presence and severity of constipation (0 = no problem, 1 = some problem, 2 = severe problem). The total possible score ranges from 0 (no constipation) to 16 (severe constipation). A score of ≥2 will trigger the use of a constipation management plan. This scale’s validity and reliability have been established [46, 47].

#### PPQ and FPQ

The nine-item PPQ measures individuals’ knowledge of cancer pain. In cases of patient/FC dyads, a similar tool, the FPQ, is used for the FC [48]. Each item is scored using a 0–10-point NRS, with an overall/average score obtained by summing the items and dividing by 9. Extensive psychometric testing of the PPQ’s and the FPQ’s English versions has established excellent validity and reliability [49, 50]. As a part of their pilot study, Koller et al. [33] translated the English PPQ/FPQ into German.

#### Self-efficacy questionnaire in cancer patients and family caregivers

The Self-Efficacy Questionnaire includes 15 items measuring perceived ability to manage specific aspects of pain on a scale ranging from 10 (very uncertain) to 100 (very certain). The questionnaire is separated in three subscales: one for pain management, one for physical function and one for other symptoms. A total score is calculated by summing the scores for each of the subscales. To assess caregivers’ confidence regarding their ability to support cancer pain management, a caregiver version of the self-efficacy scale was used. This version is identical to that applied in cancer patients except that caregivers are asked to rate how confident they are that they can support the control of patients’ cancer pain [22]. Construct validity and reliability have been established for these questionnaires [51, 52].

#### Hospital anxiety and depression scale (HADS)

The HADS, a non-diagnostic self-report screening instrument, is used to assess anxiety and depression symptomatology. Based on two 7-item subscales, each using a 4-point (0–3) Likert-type scale, a score of 11 or more (of a possible 21) on either subscale indicates significant psychological morbidity (< 8 = clinically insignificant; 8–10 = borderline; 11–21 = indicate significant psychological morbidity) [53].

#### Eastern cooperative oncology group performance status (ECOG-PS)

The ECOG-PS measures functional status, i.e., the capacity to perform a variety of activities considered normal for most people. Developed in 1960, the scale has been widely used in clinical trials and oncology practice [54]. It consists of a single 5-point scale using verbal descriptors (0 = “fully active”, 4 = “completely disabled”).

### Qualitative data

#### Data from intervention sessions

All intervention sessions are audio recorded by the IN who provides the intervention. The PhD student (SV) then listens to these audio-recordings and transcribes all relevant passages, that is, all that concern side effect management, learning processes, or FC involvement. Additionally, field notes serve as qualitative data.

#### Individual post-RCT interviews

Selected patients and FCs are interviewed by specially trained nurses who are not yet involved in the study procedures. These nurses are experienced in conducting individual interviews and have been trained by the primary investigator and the study coordinator. Following an interview guide, they ask open-ended questions to explore patients’ and FCs’ experiences with pain management and related interactions with clinicians. To better understand the degree to which the intervention meets individual needs and to identify remaining difficulties regarding pain management, participants in the IG are also asked to discuss their perceptions of the study intervention’s burdens and benefits.

Focusing on PEINCA-FAM, we hope to identify remaining difficulties concerning side effect management, to explore patients’ and FCs’ learning processes during the intervention, and to explore the FCs’ involvement and self-efficacy. Interviews, which last approximately 60 min, are audio recorded and transcribed in standard German. So that relevant new topics can be integrated in the interview guide, analyses are performed in parallel with data collection.

### Data analysis

Quantitative data will be compiled and analysed using SPSS 24.0. Data will be retrieved from the SecuTrial® database. Data will be systematically examined for out of range values and data inconsistencies. As appropriate, descriptive statistics will be calculated, including means and standard deviations for interval variables and frequencies, as well as percentages for categorical variables. An intent-to-treat analysis will be applied, using a significance level of .05 [55]. Although premature withdrawal is expected to be a random process, participants who completed the study will be compared with those who did not based on demographics, treatment group and other salient variables.

#### Data analysis for aim 1

Generalized linear mixed models will be applied to determine differences between the IG and the CG analgesic side effect developments throughout the intervention. This approach allows calculation of the main group and time effects, and of group-by-time interaction. The influence of missing data on the model results will be examined using sensitivity analysis [56]. Change scores for each patient as well as Cohen’s d will be calculated for each variable of interest.

#### Data analysis for aim 2

Our quantitative analysis will use descriptive statistics to summarize PPQ/FPQ items’ distribution characteristics. Generalized linear mixed models will be applied to determine differences between the IG’s and the CG’s knowledge of cancer pain management throughout the intervention. The influence of missing data on the model results will be examined using sensitivity analysis [46]. Change scores for each patient/FC as well as Cohen’s d will be calculated for each variable of interest.

For qualitative data analysis, transcript data will be stored and analysed in ATLAS.ti 7. Field notes and audiotapes of telephone calls, home visits and interviews will serve as qualitative data to explore both the IG’s and the CG’s the learning processes concerning knowledge of cancer pain and pain self-management. Data will be analysed via interpretive description–an approach using stepwise, systematic and iterative processing of data to arrive at a meaningful description and interpretation [57].

Information from quantitative and qualitative data collection will be combined within a mixed method matrix. Qualitative and quantitative results related to each German PPQ/FPQ item will be integrated in a final synthesis [39, 58].

#### Data analysis for aim 3

For the quantitative analysis, to determine associations between FCs’ pre- and post-intervention score changes regarding self-efficacy, knowledge of cancer pain and patients’ average and worst-pain intensity between the two groups, we will calculate Pearson correlation coefficients.

Qualitative data will be generated in the same way as for aim 2 via field notes, recordings of telephone calls, home visits and interviews. Data will be analysed by applying interpretive description [57].

Information from quantitative and qualitative data collection will be combined within a mixed method matrix and integrated in a final synthesis [39, 58].

### Handling of data

Data are handled confidentially and stored in a locked cabinet for 10 years. Data are anonymized by applying numerical IDs. The master file with patient names and associated ID numbers is kept secure. Only the study team has access to the written and electronic data. With regard to the final trial data set, only the primary investigator, the study coordinator, the PhD student and the person who enters the data will have access. In order to communicate trial results to participants, health care professionals and the public, we plan to publish results of primary and secondary outcomes. Furthermore, the study team and nurse managers of participating university hospitals will be informed about study results via scientific presentations in various settings.

## Discussion

This study is the first to evaluate the effects of a psychoeducational intervention to reduce analgesics’ side effects and to explore the involvement of FCs in pain management in a sample of Swiss cancer outpatients and their FCs. As with other chronic illnesses, cancer places new demands on patients and their FCs to manage their care; however, to do so effectively requires specialized skills. Therefore, to achieve adequate pain and side effect self-management, cancer patients and their FCs require educational interventions. Their self-management support should focus on enhancing patients’ and FCs’ pain management knowledge and self-efficacy, while improving their use of pain and side effect relief strategies [24, 59, 60].

This RCT involves the evaluation of a multicomponent self-management support intervention that combines monitoring of pain, side effects and medication based on nurse coaching via weekly in-home or telephone visits, skill building, and information provision. Compared to usual care, self-management support is expected to result in improved control of pain and analgesics’ side effects, as well as enhanced self-efficacy and knowledge [61].

Previous qualitative studies on home cancer pain management have showed that cancer patients have to manage diverse analgesics’ side effects, which is an ongoing multidimensional process [12, 32]. The complexity of this process means that, in addition to strategies and techniques for effective pain management, it is important to buttress both patients’ and FCs’ self-management and self-efficacy via an effective, holistic side effect management regimen [12, 62].

The proposed psychoeducational intervention, the PRO-SELF © Plus PCP, differs from previous attempts in its interactive, multicomponent approach: (1) Following evidence-based guidelines, a comprehensive pain assessment–including rating scales alongside patient reports of pain qualities, side effects, treatments and satisfaction with pain relief–is performed first at baseline, then at each contact [63]. (2) Because the side effects of cancer treatment can severely impact pain self-management, we focus particularly on information and coaching to manage those effects [7, 46]. (3) Based on an academic detailing approach, this intervention focuses on adult learning principles, providing not only key information but also positive reinforcement to enhance baseline knowledge. Furthermore, it stimulates the learner as an active partner [42, 43]. (4) This intervention will train cancer patients and their FCs to improve their own self-efficacy and self-management by keeping a diary, setting goals and establishing a symptom management plan to achieve those goals. Overall, the described combination of quantitative and qualitative data will illuminate the proposed intervention’s efficacy, enhancing the interpretation of its results and improving its eventual implementation [64].

Previous evidence indicates that, while the recruitment of oncology patients is rarely easy, it can be especially challenging for studies involving symptom management [65, 66]. Obstacles to recruitment can include the characteristics of the patients themselves, the recruiting clinician, the trial centre, the trial organization and the trial design [67]. To increase enrolment, it is important to provide detailed information about the study procedures and the advantages and disadvantages of the intervention [68]. The study coordinator visits the participating departments regularly. Study information and news of the ongoing process are shared regularly between study team members; and the steering committee continuously supervises the study procedures.

Further, previous research experiences have taught us that, in this already strained population, minimizing participation burden will likely increase enrolment while remaining efficacious [65, 69]. Therefore, we have reduced the original 10-week study period [33] to 6 weeks, and stress maximum flexibility in scheduling home visits.

This study’s results will contribute to the understanding of interventions designed to improve side effect self-management, knowledge and self-efficacy in cancer outpatients and their FCs. If efficacious, the proposed intervention could be implemented in clinical practice to reduce pain and analgesics’ side effects, while enhancing patients’ and their FCs’ pain management skills and knowledge.

## Abbreviations

CAS: Constipation Assessment Scale
CG: Control group
CTU: Clinical Trial Unit
ECOG-PS: Eastern Cooperative Oncology Group Performance Status
FCs: Family caregivers
FPQ: Family Pain Questionnaire
GCP: Good Clinical Practice
HADS: Hospital Anxiety and Depression Scale
ICH: International Council for Harmonization of Technical Requirements for Pharmaceuticals for Human Use
IG: Intervention group
NRS: Numeric Rating Scale
NSAIDs: Nonsteroidal Anti-Inflammatory Drugs
PPQ: Patient Pain Questionnaire
PRO-SELF © Plus PCP: PRO-SELF © Plus Pain Control Program
RCT: Randomized controlled trial
SCT: Social Cognitive Theory
SMM: Symptom Management Model
TSM: Theory of Symptom Management
WHO: World Health Organization
